# Characteristic analysis of volatile avalanche diode threshold switching for bionic nerve synapse applications

**DOI:** 10.1038/s41598-021-00594-y

**Published:** 2021-10-26

**Authors:** Yang Wang, Zeyu Zhong, Xiangliang Jin, Yan Peng, Jun Luo

**Affiliations:** 1 Department of College of Physics and Electronic Science, School of Hunan Normal University, Changsha, 410081 China; 2 Department of College of Mechatronic Engineering and Automation, School of Shanghai University, Shanghai, 200444 China

**Keywords:** Neuroscience, Engineering

## Abstract

The combination of biological neurology and memristive theory has greatly promoted the development of neuromorphic computing. To build a large-scale artificial intelligence alert system, the exploration of bionic synapses compatible with standard processes has become an urgent problem to be solved in the next step. In response to the above application requirements, this paper proposes a volatile avalanche diode threshold switching (VADTS) that is fully compatible with standard semiconductor technology to simulate the various functions of the synapse. Technology computer-aided design device-level simulation can verify the bionic principle of VADTS. The function of VADTS's bionic synapse was verified by the experimental test platform. The results show that under the action of the excitation signal (11.25 V), the device can continuously change from a high-resistance state to a low-resistance state. When the excitation signal is lower than the threshold, VADTS presents a “no adaptation” state of nerve synapses. When the excitation signal is higher than the threshold and changes continuously, the current changes along with the amplitude of the excitation signal, similar to the “sensitization” state of the nerve synapse.

## Introduction

Artificial intelligence will overturn the traditional information age and cause a new scientific and technological revolution. Bionic robots with artificial intelligence algorithms greatly facilitate human life. The artificial alert system can guide the robot to respond in time to changes in the working environment, thereby greatly improving the intelligence and accuracy of the robot. The threshold switching characteristics, nonlinear effects, and logic computing capabilities of the memristor^1–3^ not only provide design ideas for the storage-computing integrated chip architecture^4^, but also provide solutions for artificial intelligence bionic applications^5^. As a key sensory receiver, the nerve synapse can efficiently recognize a variety of harmful stimuli. When a stimulus signal comes, the synapse generates a warning signal and transmits it to the brain's nerve center to initiate a stress response. However, when the amplitude of the pulse signal generated by the external stimulus is lower than the threshold, the nerve synapse will assume a “no adaptation” state. When the amplitude of the pulse signal generated by the external stimulus is higher than the threshold, as the signal amplitude increases, the warning signal generated by the synapse will also change with the change of the excitation signal, that is, the “sensitization” state^6,7^. Robots equipped with nerve synapse bionic devices will further advance into the field of intelligence.

The simulation of biomimetic nerve synapses requires devices to have good threshold switching characteristics and nonlinear effects. The resistance state of the devices can be flexibly adjusted by excitation signals of different amplitudes, so as to realize the basic functions of synapses. With the continuous development of memristive theory, more and more scientists have conducted research on memristors^8–10^. R. Singh et al. designed a solid electronic synapse with a heterogeneous structure. The device exhibits memristive characteristics under the excitation of continuous signals. Moreover, because the NiO layer inside the device can enhance the trapping ability of charge carriers, the stability and switching uniformity of the memristor can be greatly improved. This research provides a design idea for low-cost bionic synapses^11^. X. Liang et al. prepared a thin-film memristor based on a single crystal thin-film through an oxygen annealing process. Through the above process method, the robustness of the device can be significantly improved. The impulse test shows that the device successfully simulates the time-related plasticity of the bionic synapse, and can be used as an electronic synapse for brain-like neuromorphic calculations^12^. LX. Hu et al. carried out the study of all-optical control (AOC) analog memristor for neuromorphic computing. The material of the device is InGaZnO, which realizes the non-volatility of the memristor through a mediation tuning mechanism. AOC memristors can simulate the plasticity of spike timing, and further provide potential value for efficient optoelectronic smart applications^13^. With the rise of bionic engineering technology, the research of spike neural networks (SNN) is gradually advancing. KL. Long et al. researched SNN circuits based on a single electron transistor (SET) combined with a memristor. The relevant bionic characteristics of the SNN circuit are realized by establishing the PSPICE model^14^. S. Kim et al. proposed a memristor based on SiNx material to simulate the switching synaptic characteristic of resistance. The memristor is compatible with complementary metal oxide semiconductor technology (CMOS), but further process modifications are required to realize neuromorphic devices with biological synaptic functions^15^. From the perspective of simulation, VK. Rai et al. used nano-scale nonvolatile memristors to simulate biological synapses. By comparing with traditional CMOS bionic synapses, the device proposed by the author achieves a higher peak frequency and lower power consumption in the simulation results. It provides a reference for the simulation research of nanometer memristors^16^. The above-mentioned research on memristors has greatly promoted the development of bionic electronic synapses. Through new materials, electronic synapses designed by nanotechnology have better bionic characteristics^17–21^. However, artificial intelligence chips have put forward large-scale array integration requirements for devices with threshold switching. Therefore, devices that are not fully compatible with microelectronics processes are difficult to achieve integration in a short period time.

This paper presents a silicon-based volatile avalanche diode threshold switching. VADTS has a stable threshold voltage and nonlinear characteristics. The current gain after the device is turned on is high, and hence it can be used for the simulation of bionic synapses. Through principle analysis, simulation verification, and experimental test, the results show that VADTS can achieve the “no adaptation” and “sensitization” characteristics of bionic nerve synapses. Moreover, the biggest advantage of above device is that it is fully compatible with standard CMOS technology. No additional mask modification is required. And large-scale on-chip integration of bionic nerve synapses can be realized through arraying. The VADTS provides a low-cost, simplified volatile threshold switching structure prototype for the future artificial intelligence alert system.

## Structure and principle of VADTS

**Figure 1:**
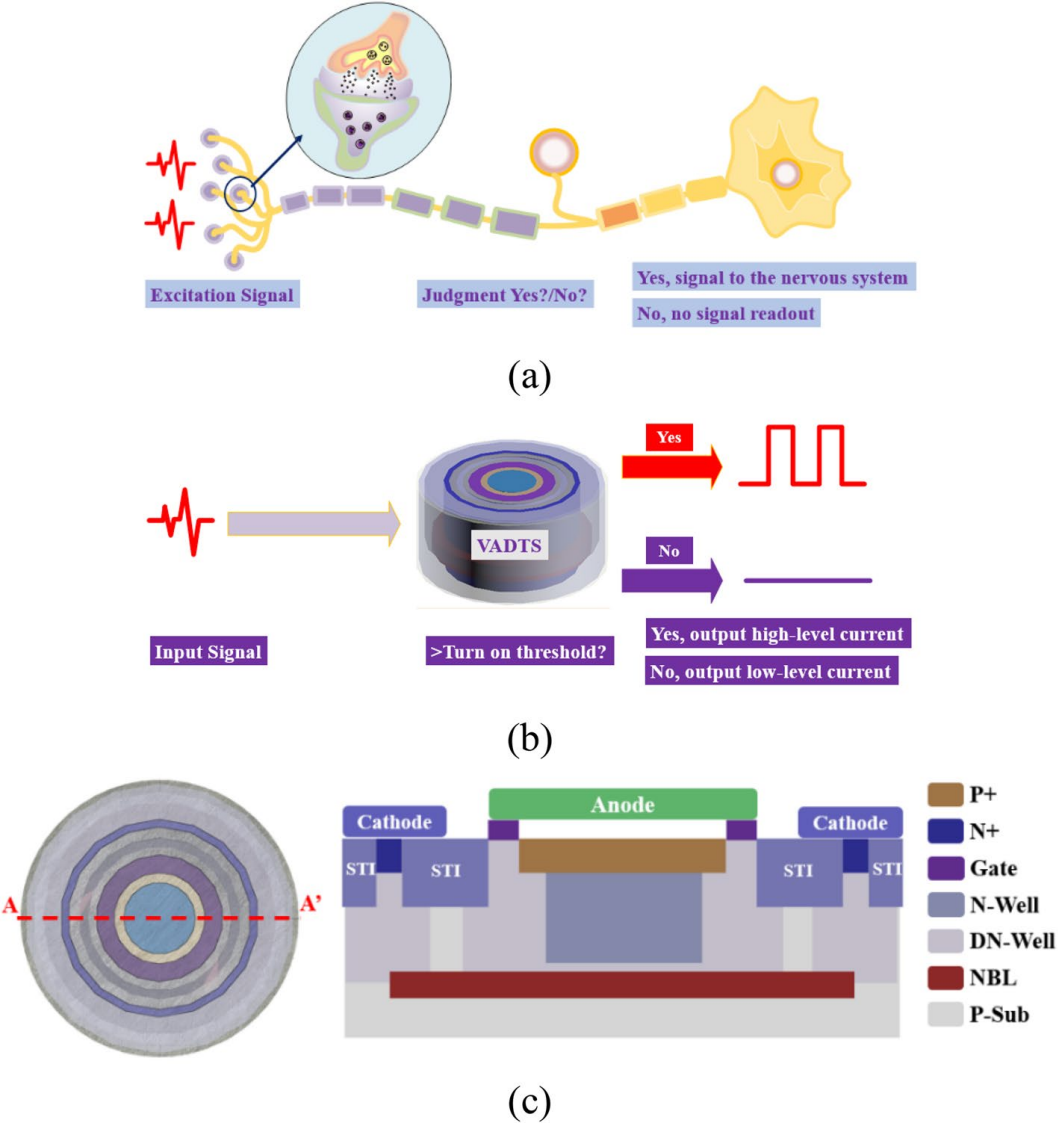
(**a**) The signal transmission mechanism of the nerve synapse. (**b**) The signal transmission mechanism of VADTS bionic nerve synapse. (**c**) The cross section of VADTS device.

There are a large number of nerve synapses in the human body. Synapses are the key part of transmitting information between neurons. There are more synaptosomes at the axon ends of neurons. These synaptosomes can contact the cell bodies or dendrites of multiple neurons to form synapses. As shown in Fig. 1a, synapses can identify stimulus signals. When the stimulus signal comes, the synapse generates a warning signal and transmits it to the central nervous system of the brain to make a quick stress response. The unique threshold switching characteristics of avalanche diode can simulate the biological functions of synapses, as shown in Fig. 1b. When an external stimulus signal comes, the VADTS first compares the amplitude of the stimulus signal with the threshold of the device. If the stimulus signal is lower than the device threshold, the VADTS judges the signal as a “harmless signal”, and the device remains closed at this time, showing a “no adaptation” state. If the stimulus signal is higher than the device threshold, the VADTS judges the signal as a “harmful signal”, and the device is turned on at this time, showing a “sensitization” state.

The structure of the device that simulates the nerve synapse is shown in Fig. 1c. The structure prototype of VADTS is an avalanche diode. When the amplitude of the excitation signal is lower than the threshold voltage, the device is in the off state, and the output current at this time is the reverse leakage current of the diode. When the amplitude of the excitation signal is higher than the threshold voltage, the electron holes inside the device collide with atoms in the depletion zone under the action of a strong electric field, causing the valence electrons to break free from the covalent bond and generate free electron–hole pairs. Through a similar chain reaction, the number of carriers in the depletion region increases exponentially. At this time, the output current of the device will be much greater than its reverse leakage current. The threshold voltage of VADTS is mainly determined by the avalanche breakdown voltage of P+ and N-Well. When the device is turned on, the ring-shaped polysilicon gate at the edge of the P+ isolates the avalanche multiplication region from the shallow trench isolation (STI) and suppresses the carrier trapping effect caused by the material energy level defects. Thus, the mis-trigger probability of the device is greatly reduced, and the accuracy is improved. VADTS forms a dummy guard ring through DN-Well. Due to the Gaussian concentration gradient of DN-Well, the electron concentration at the edge of P+ is much lower than that in the central area of P+ , which further promotes the electric field force to concentrate on P+ and N-Well of the plane PN junction. The existence of the guard ring allows the device to work in avalanche mode for a long time, improving the stability of the device. At the bottom of the VADTS, the N-type heavily doped buried layer (NBL) can isolate the noise carriers of the P-type substrate, keeping the dark current of the device at the nA level and effectively improving the transparency of the VADTS. When the voltage excitation signal above the threshold comes to the cathode of the device, VADTS is in the reverse bias state (low-resistance state). The current of the device is multiplied to the μA (mA) level under the action of the avalanche effect, resulting in a significant current readout. When the excitation signal disappears, VADTS returns to the off state (high-resistance state), and the current of the device drops to nA level. In summary, under the action of the stimulus signal, the resistance of the VADTS can be continuously switched between the high-resistance state and the low-resistance state, achieving threshold switching characteristics. Moreover, whether it is a continuous signal or a discrete signal, the current of the device continuously changes with the voltage, thereby realizing basic functions of the nerve synapse.

## Two-dimensional simulation and discussion

**Figure 2:**
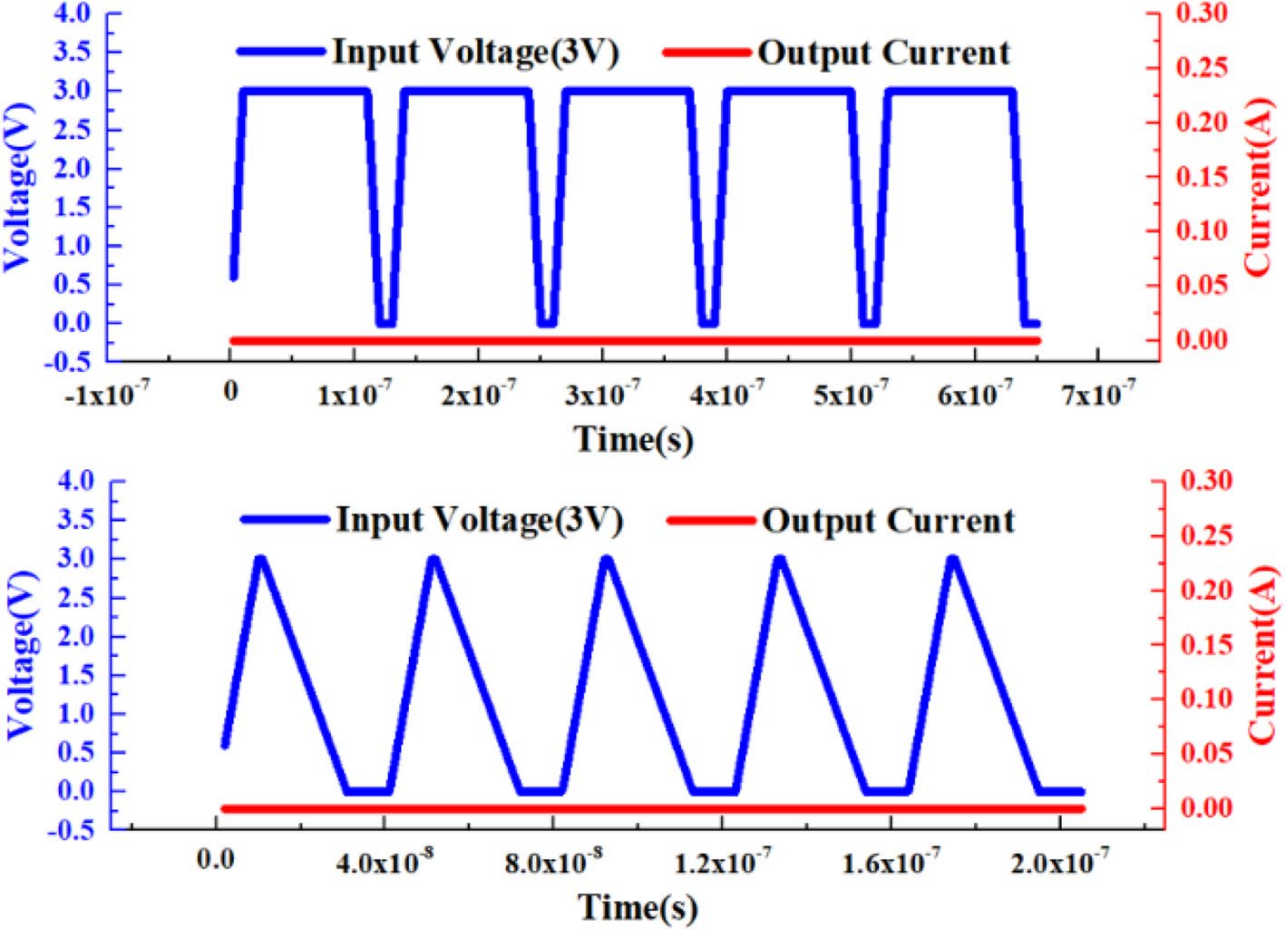
VADTS simulation voltage–time curve and simulation current–time curve (3 V).

**Figure 3:**
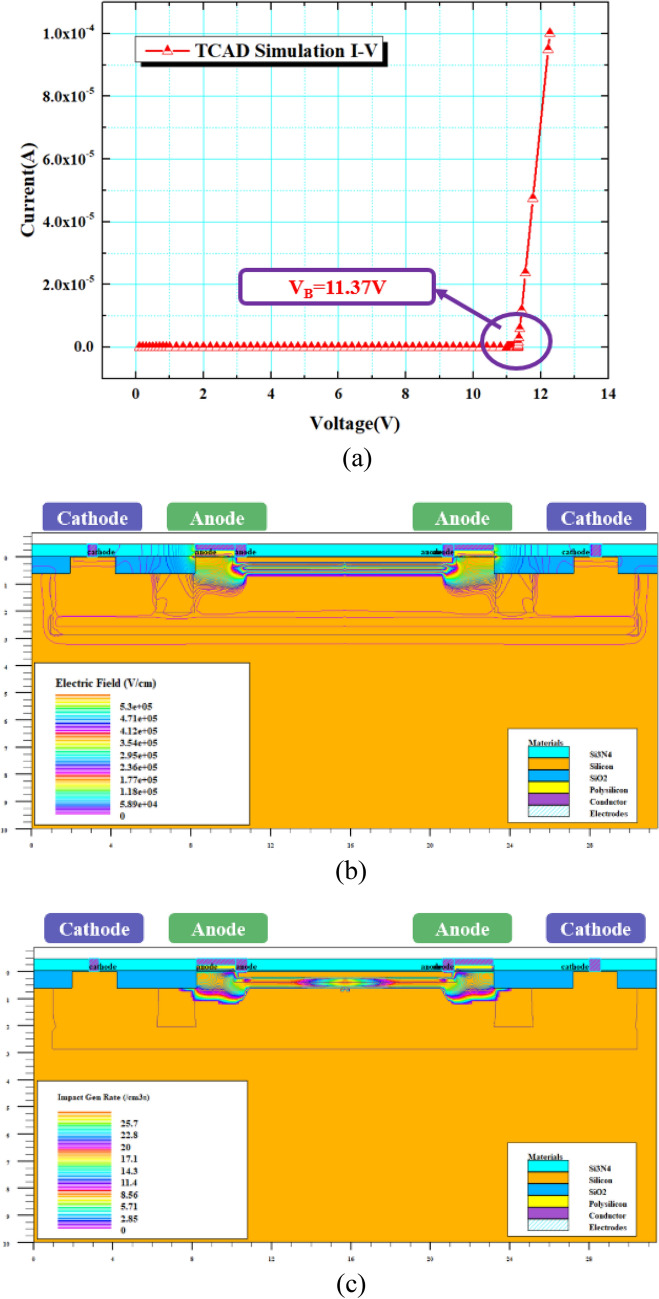
(**a**) The simulation I-V curve of VADTS. (**b**) The electric field distribution after VADTS is turned on. (**c**) The impact ionization distribution after VADTS is turned on.

Slivaco is used to perform one-dimensional and two-dimensional electrical simulations on VADTS, as shown in Fig. 2. When a 3 V square wave signal or pulse signal is applied to the cathode of the VADTS, the device is in the off state at this time and no obvious current is read out. Since the device threshold voltage is much higher than the external excitation signal, the “no adaptation” state of the device can be verified by setting the excitation signal with a smaller amplitude. In order to verify the basic principles of VADTS, the I-V characteristics, electric field distribution, and impact ionization distribution of the device were obtained by DC simulation, as shown in Fig. 3a–c. The simulation result shows that the avalanche breakdown voltage of the device is 11.37 V. The polysilicon ring gate effectively isolates the avalanche multiplication region and STI. The electric field effect is mainly concentrated in the reverse-biased PN junction of the P+ and N-Well of the device anode. Through analyzing the results of impact ionization distribution, it can be found that the DN-Well guard ring ensures that the VADTS works stably in avalanche mode, and the edge breakdown effect of the PN junction will not occur in advance. The above simulation verifies the working principle of VADTS.

**Figure 4:**
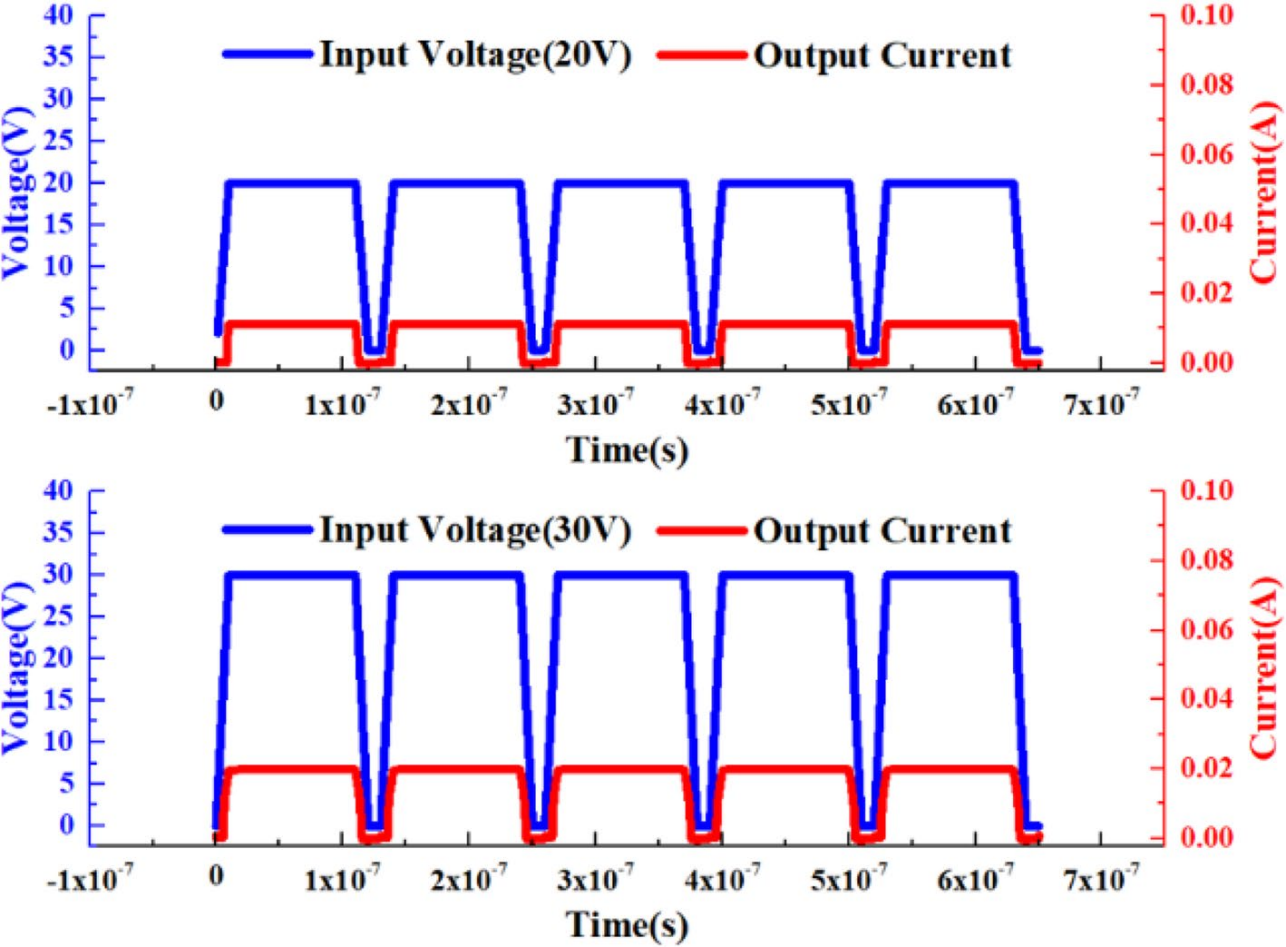
Simulation voltage–time curve and simulation current–time curve of VADTS under the excitation of square wave signal (20 V, 30 V).

**Figure 5:**
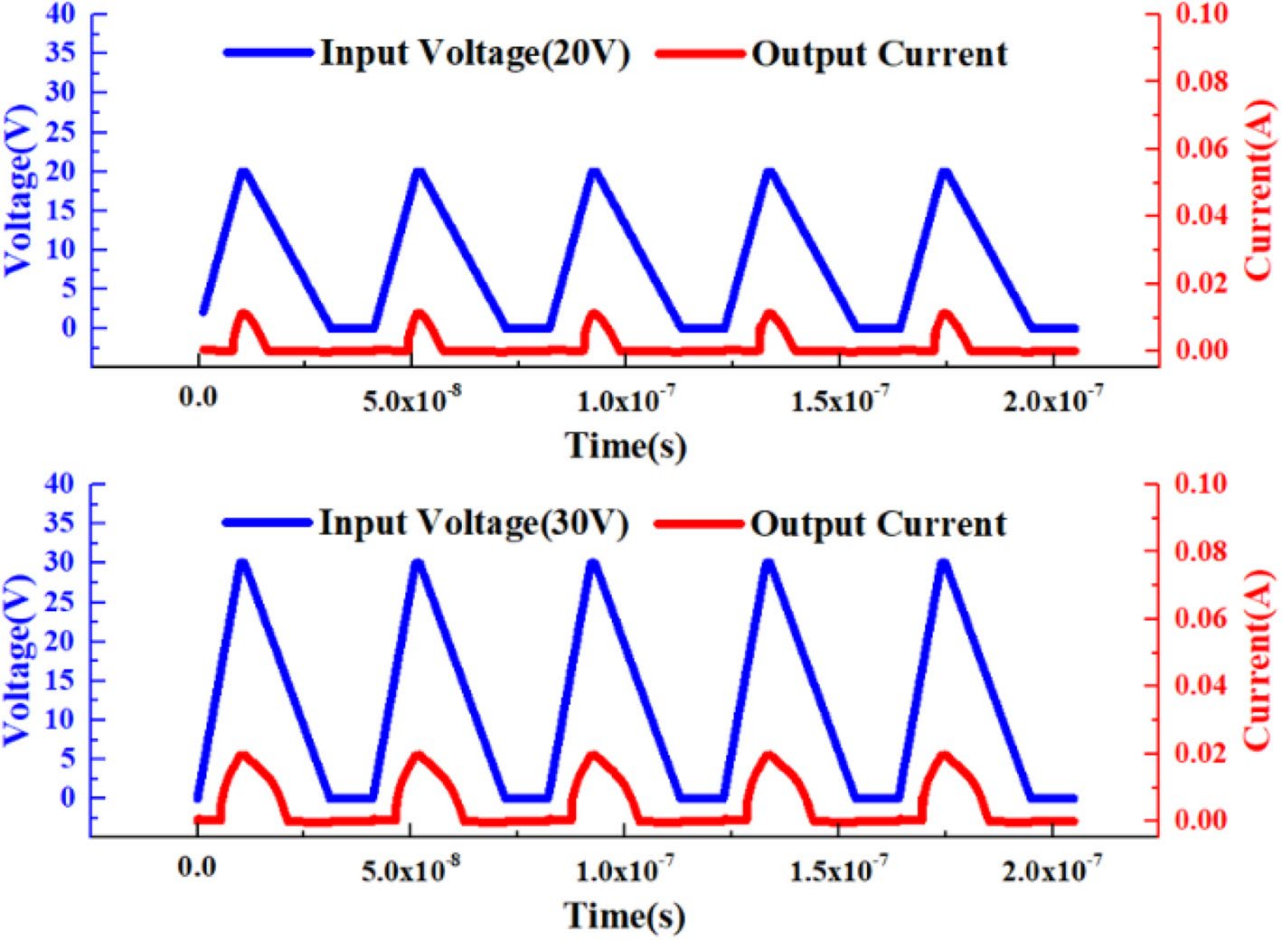
Simulation voltage–time curve and simulation current–time curve of VADTS under the excitation of pulse signal (20 V, 30 V).

In order to further verify the nerve synapse function of the device, an excitation signal higher than the threshold voltage of VADTS was used to characterize the “sensitization” state of the device. As shown in Fig. 4, the amplitude of the applied excitation signal is 20 V. When the square wave voltage signal is at a high level, the current of the VADTS will change with the change of the voltage amplitude and rise rapidly to 0.015A. When the square wave voltage signal is at a low level, the VADTS current drops to nA. At this time, by further increasing the voltage amplitude (30 V) of the external excitation signal, it can be found that in the same period, when the voltage is at a high level, the current instantly surges to 0.02A. When the voltage drops to a low level, the current drops to nA. The simulation of the square wave excitation signal cannot fully verify the bionic function of VADTS. Therefore, transient voltage pulses are used to verify the sensitivity of the device. As shown in Fig. 5, when the amplitude of the transient voltage pulse increases, the transient current pulse of the device increases with the increase in voltage, and presents continuous changes in the same period. The simulation results of square wave signal and impulse signal proved that VADTS can realize the basic function of nerve synapse. TCAD simulation is limited by the process level and cannot quantitatively predict the synaptic characteristics of the device, but it can qualitatively describe the bionic mechanism of VADTS.

## Experimental results and discussion

**Figure 6:**
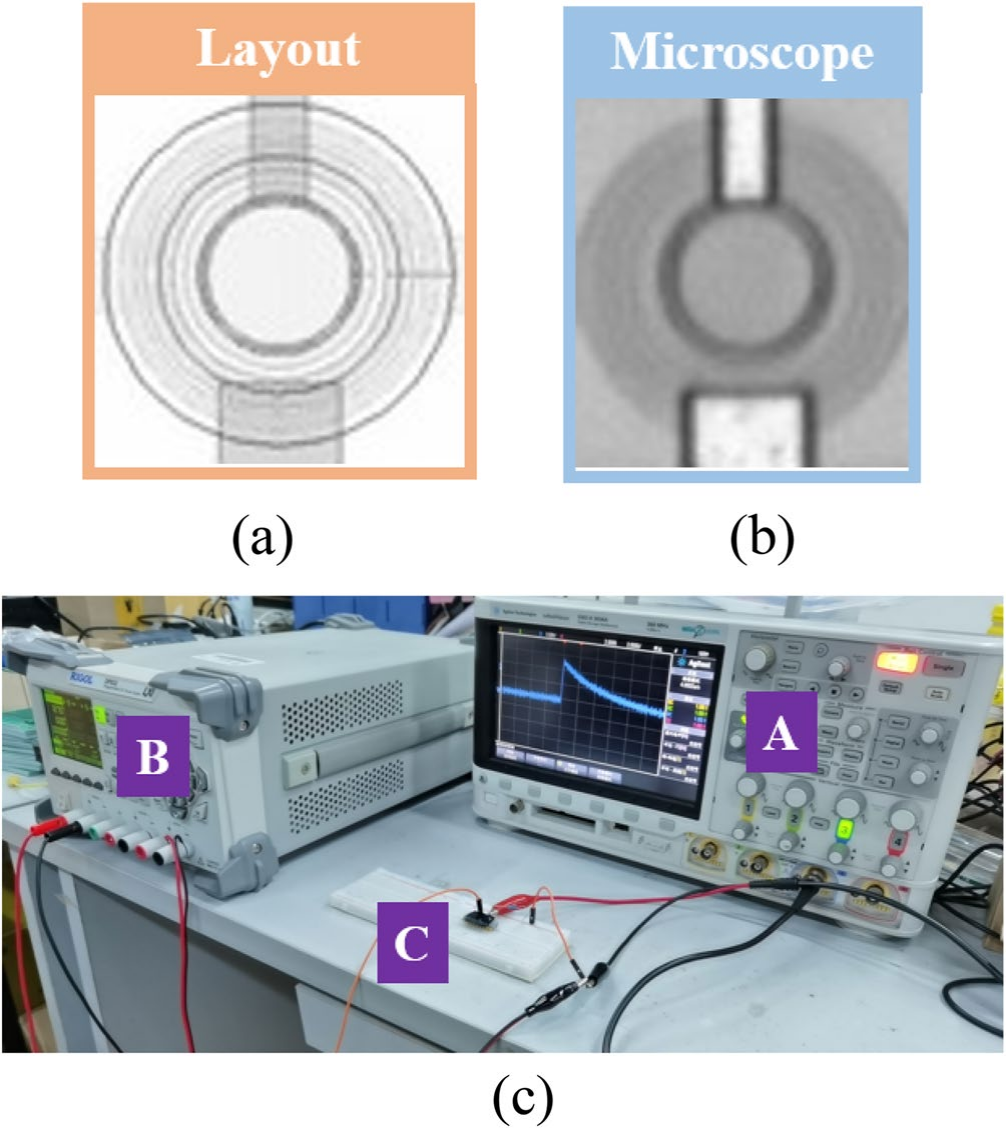
(**a**) The layout of VADTS. (**b**) The microscope image of VADTS. (**c**) The experimental platform of VADTS (A: oscilloscope, B: power supply, C: volatile avalanche diode threshold switching).

VADTS is manufactured using a 0.18 μm BCD process. The device layout is circular, which effectively reduces the edge breakdown effect of the avalanche multiplication zone so that the device can obtain a uniform electric field distribution. The bionic synapse test of VADTS was carried out by establishing an experimental platform. Firstly, the “no adaptation” state of VADTS is verified. Then the threshold voltage of the device is obtained by the DC test. When the device is turned on, the voltage and current of the VADTS are extracted at the same time. The threshold switching characteristics of VADTS can be verified by fitting the hysteresis loop^22–24^. Finally, the characteristic relationship between the continuous voltage pulse and the current pulse verifies the “sensitization” characteristics and sensitivity of VADTS. The device layout, microscope image and experimental platform are shown in Fig. 6a–c respectively.

**Figure 7:**
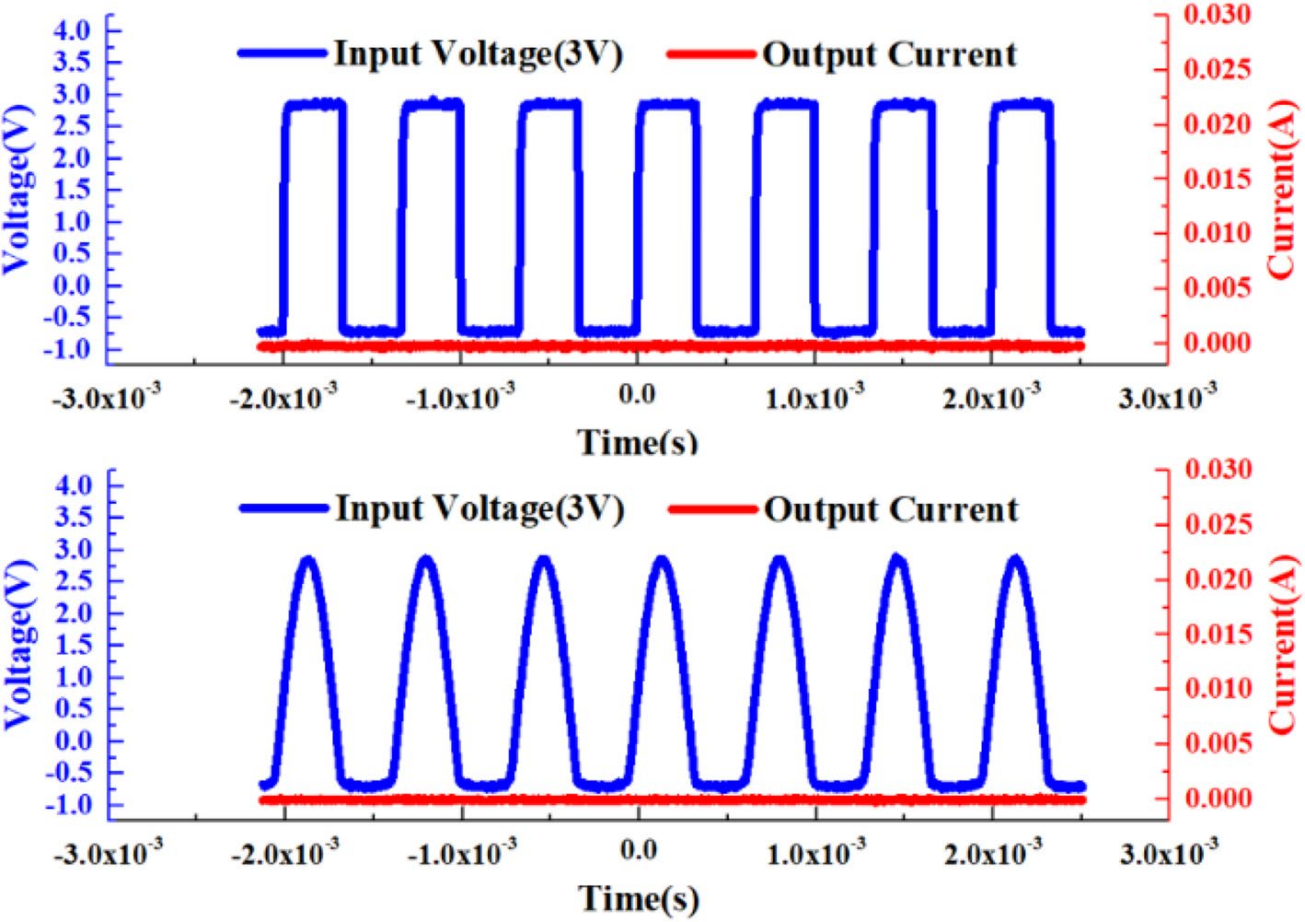
Test voltage–time curve and test current–time curve of VADTS under the excitation of square wave signal and pulse signal (3 V).

**Figure 8:**
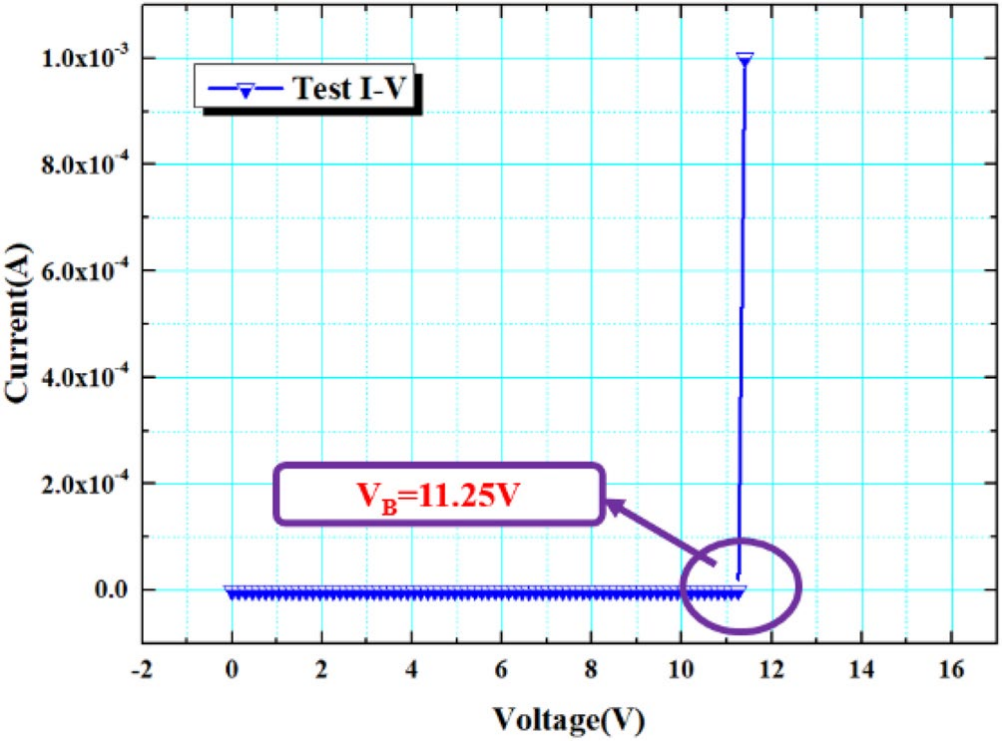
The test I-V curve of VADTS.

**Figure 9:**
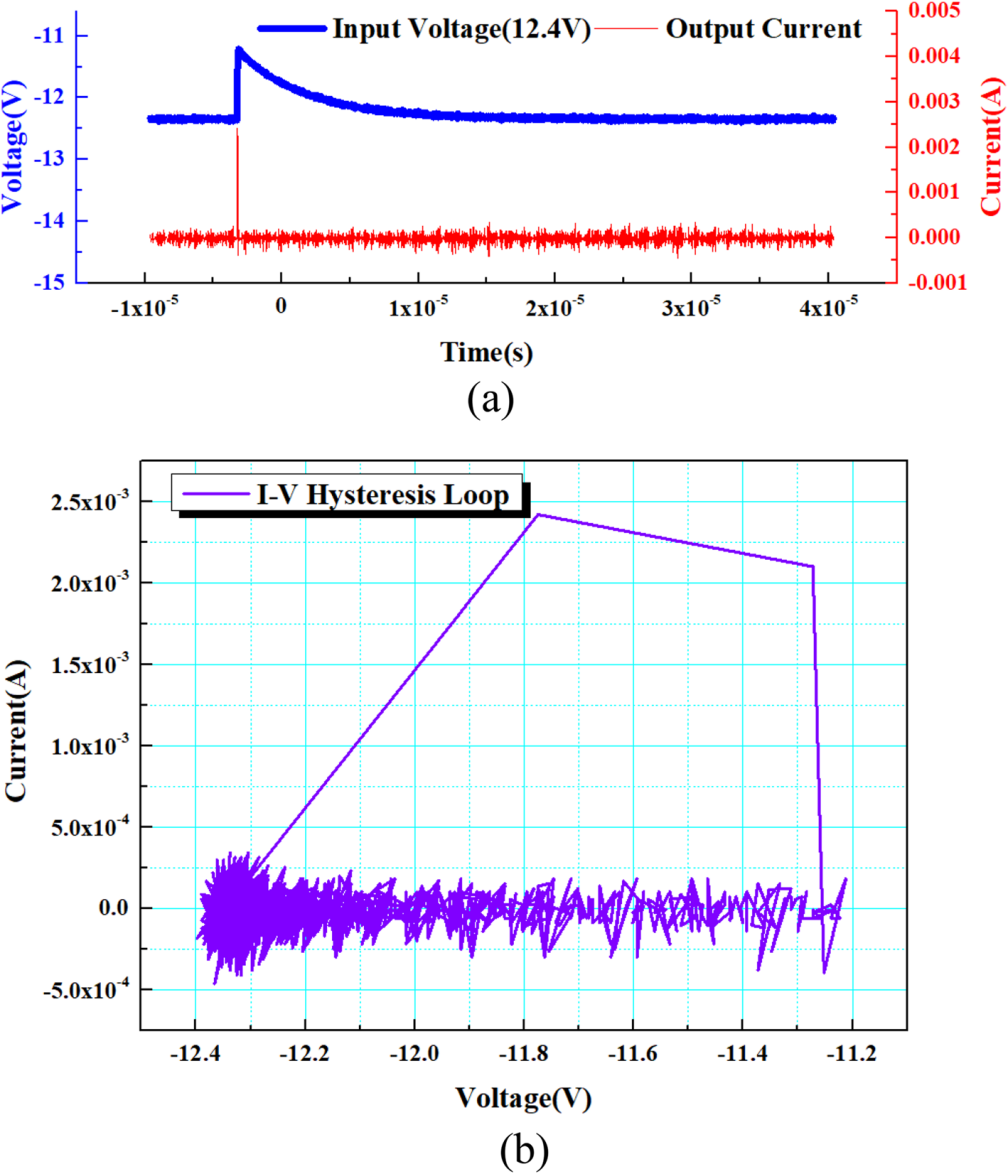
(**a**) Test voltage–time curve and test current–time curve of VADTS under the excitation of pulse signal. (**b**) Test hysteresis loop of VADTS.

When a square wave signal or a pulse signal with an amplitude of 3 V is applied to the cathode of the device, the “no adaptation” characteristic test of VADTS is shown in Fig. 7 (when the amplitude of the excitation signal is less than the threshold voltage, the device is in the off state. The current does not change with the change of the voltage, verifying the “no adaptation” state of VADTS). At this time, VADTS is in reverse-biased mode, but the device is still in the off state because the excitation signal amplitude at both ends of the electrode is far below the threshold. The current at both ends of VADTS is always at the nA level, and no obvious current pulse is generated. The IV test curve of the device is shown in Fig. 8. The result shows that its avalanche breakdown voltage is 11.25 V. When VADTS works in avalanche mode, the current will quickly double to the mA level. The current of the device in the off state is nA, and the current in the on state is μA (mA). The difference between the current amplitude can be used as the judgment condition of the low-resistance state and the high-resistance state of the device, and it is also a necessary condition to satisfy bionic nerve synapse. In order to verify the threshold switching characteristics of VADTS, a voltage pulse with an amplitude of 12.4 V was applied to both terminals of the device. The test result is shown in Fig. 9a (since a 100Ω resistor is connected in series during the device test, the current value of the device is characterized by extracting the voltage change of the resistor. Therefore, the voltage signal extracted under the reverse bias of the device is negative). Through the test results, it can be found that when the excitation pulse voltage signal rises to a peak, the current of the VADTS increases with the increase in the voltage. And due to the influence of the junction capacitance of the device, the current pulse lags behind the voltage pulse. By extracting the current pulse and voltage pulse of the device at the same time, the hysteresis loop is fitted and the threshold switching characteristics of VADTS can be verified, as shown in Fig. 9b.

**Figure 10:**
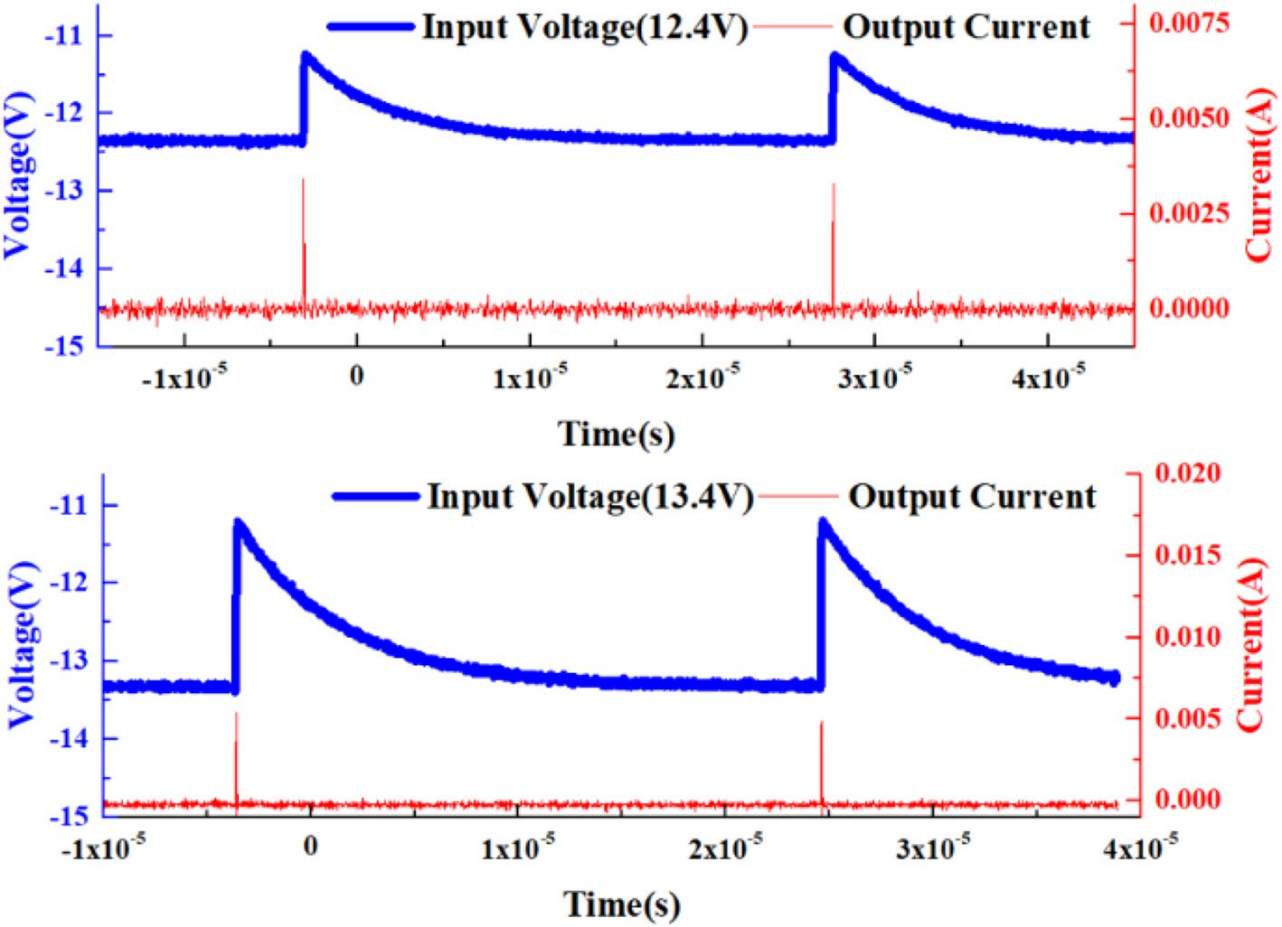
Test voltage–time curve and test current–time curve (12.4 V, 13.4 V) of VADTS under the excitation of multisegment pulse signals.

**Figure 11:**
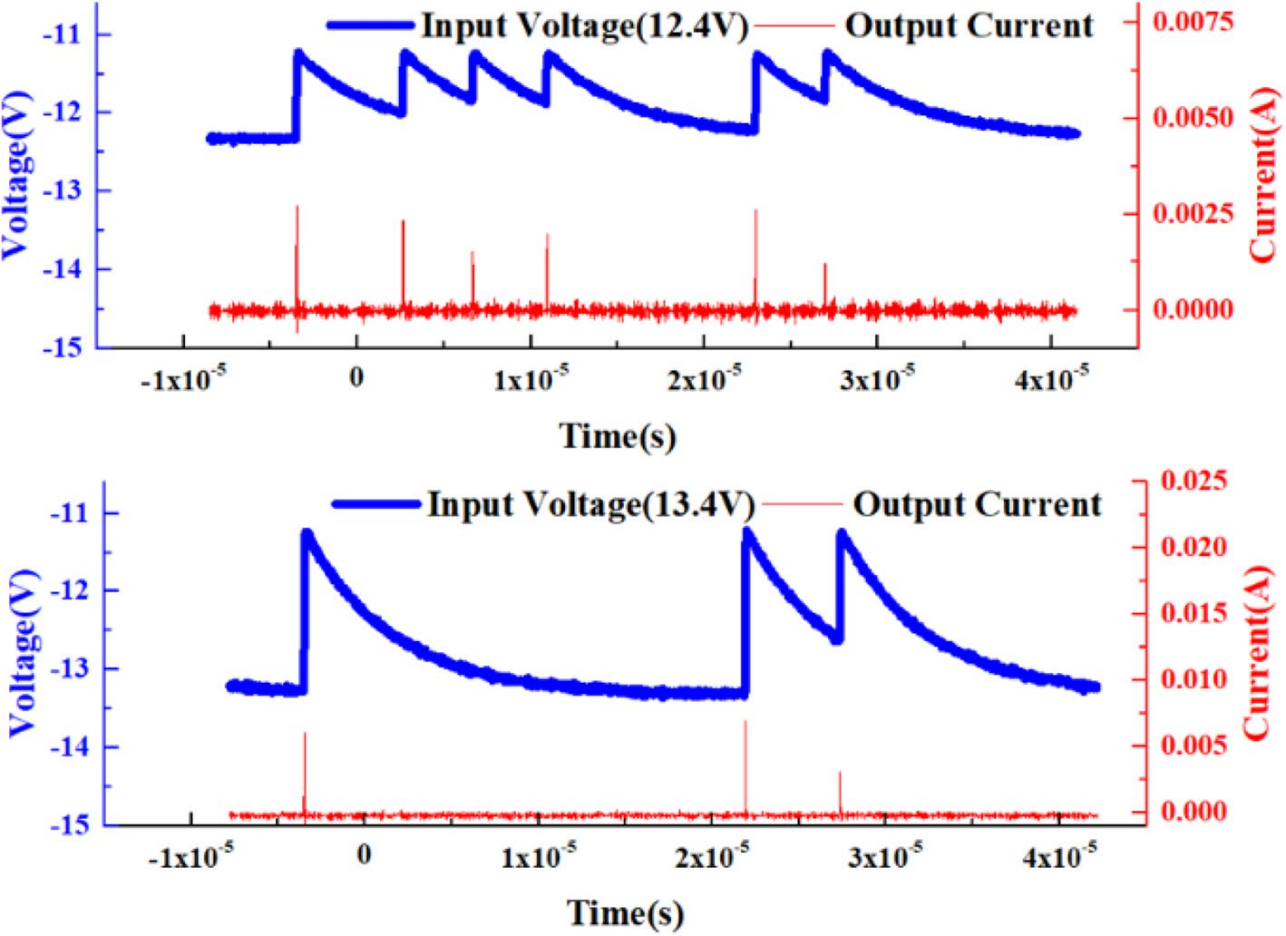
Test voltage–time curve and test current–time curve (12.4 V, 13.4 V) of VADTS under the excitation of random continuous pulse signals with short intervals.

The “sensitization” characteristic of VADTS can be verified by pulse voltage excitation signals of different amplitudes, as shown in Fig. 10. When the amplitude of the pulse excitation voltage signal increases from 12.4 to 13.4 V, the current of the device increases from 2.5 × 10^−3^ to 5 × 10^−3^ A. Moreover, under the excitation of multiple pulse signals at intervals of time, the current of the device still changes continuously with the change of the voltage. In order to further verify the sensitivity of VADTS, a random continuous pulse with a short interval is set and applied to the cathode of the device. The test results are shown in Fig. 11. On the premise that the previous pulse signal has not disappeared, a voltage pulse of the same amplitude is continuously applied. At this time, VADTS can still recognize the change of the superimposed pulse signal, thereby generating a current pulse similar to the voltage pulse change. When the excitation pulse voltage amplitude of VADTS changes from 12.4 to 13.4 V, the device can still maintain good sensitivity. Therefore, according to the principle analysis of VADTS, simulation verification and test results show that the device can realize basic functions of the bionic nerve synapse. At present, artificial intelligence bionic applications are constantly developing towards low power consumption. As the front-end device of the biomimetic synapse system terminal, VADTS can be used to judge, receive relevant signals and further transmit signals to the back-end circuit for data processing. Based on this structure, a device with a lower threshold voltage can be realized in a low-voltage process, so as to conduct research on the on-chip integration of the artificial intelligence alert system.

## Conclusion

In this paper, a volatile threshold switching is designed based on the semiconductor avalanche mechanism, which is used to simulate basic functions of the nerve synapse. TCAD simulation is used to verify the basic principles of VADTS. The basic electrical parameters of the device were obtained by building an experimental platform. The test results show that under the premise that the excitation signal is lower than the device threshold. VADTS can achieve the “no adaptation” characteristics of the bionic synapse. When the amplitude of the excitation signal exceeds the threshold of the device, the transient voltage and transient current of the VADTS are extracted at the same time, and the hysteresis loop is fitted to verify the threshold switching characteristics. The device can continuously swtich between a high resistance state and a low resistance state. Under the excitation of pulse voltage signals of different amplitudes, VADTS can realize the “sensitization” characteristics of bionic synapses. In addition, the sensitivity of the device is further verified by multiple voltage pulses with a period time and continuous superimposed pulses with a short period time. In summary, VADTS compatible with standard microelectronic technology can realize basic functions such as signal judgment and signal reception of the bionic nerve synapse and meet the requirement of large-scale on-chip integration of artificial intelligence alert systems. Based on the design principle of VADTS, low-power applications of bionic electronic synapses can be realized through low-voltage technology.
